# Reply: *EGFR* alterations and response to anti-EGFR therapy: is it a matter of gene amplification or gene copy number gain?

**DOI:** 10.1038/bjc.2011.571

**Published:** 2011-12-20

**Authors:** A Ålgars, M Lintunen, O Carpén, R Ristamäki, J Sundström

**Affiliations:** 1 Department of Oncology and Radiotherapy, Turku University Hospital, Hämeentie 11, Turku PO Box 52, FIN-20521, Finland; 2 MediCity Research Laboratory, University of Turku, Tykistökatu 6 A, Turku FIN-20520, Finland; 3 Department of Pathology, University of Turku, Kiinamyllynkatu 10, Turku FIN-20520, Finland; 4 Department of Pathology, Turku University Hospital, Kiinamyllynkatu 10, Turku FIN-20520, Finland

**Sir,**

We wish to thank Sesboüé *et al* (2012) for their interest in our study on the prediction of anti-EGFR therapy in colorectal cancer (CRC) (Ålgars *et al*, 2011). While, as they discuss, the mechanism of gene copy number (GCN) alterations may affect the response to targeted treatments, this question was beyond the focus of our work. Our aim was to test a simple hypothesis: does EGFR immunohistochemistry (IHC)-guided silver *in situ* hybridisation analysis predict treatment response better than previously used methods. Our hypothesis was based on the fact that EGFR expression in CRC, as examined by IHC, is heterogenous within tumours (Moroni *et al*, 2005; Ålgars *et al*, 2011). Thus, *EGFR* GCN (or Chr-7 polysomy) analysis from areas with highest IHC intensity might reflect the tumour's sensitivity to anti-EGFR Abs better than unguided fluorescence *in situ* hybridisation analysis. We correlated the results with three different clinical parameters; clinical benefit, as evaluated by RECIST criteria, progression-free survival (PFS), and overall survival (OS). The results showed that in unselected or *KRAS* wild-type (WT) tumours, *EGFR* GCN increase (cutoff 4.0) significantly predicts outcome by all three parameters. The mean PFS in the *KRAS* WT/*EGFR* GCN high group was three times longer than in the *KRAS* WT/*EGFR* GCN low group, and the OS of the *KRAS* WT/*EGFR* GCN high patients was four times longer than OS of the *KRAS* WT/*EGFR* GCN low patients.

The *EGFR*/Chr-7 ratio did not in our analysis have any predictive value. While 51 out of 78 tumours had an *EGFR* GCN over the cutoff value 4.0, only two of them had an *EGFR*/Chr-7 ratio >2 indicating that pure *EGFR* amplification in CRC is a rare event (at least when evaluated by our methods). Thus, *EGFR* GCN increase in association with Chr-7 polysomy appears to be the prevalent pattern, which is associated with responsiveness to anti-EGFR treatment. Sesboüé *et al* (2012) site four studies to support their claim that only ‘true’ *EGFR* amplification would be meaningful for the treatment response. Of these studies, Cascinu *et al.* correlated *EGFR* GCN changes to response with EGFR small molecular inhibitor, gefitinib, which is not used in CRC (Cascinu *et al*, 2008). One of the other three studies lacks information of the *KRAS* status of the tumours (Razis *et al*, 2008), and only one of these three studies correlated *EGFR* alterations with time to progression (Razis *et al*, 2008), whereas the other two studies assessed merely the response rates to anti-EGFR therapy (Moroni *et al*, 2005; Frattini *et al*, 2007). In our understanding, none of the studies therefore support the conclusion of Sesboüé *et al* (2012). As a final note, the dogma of the importance of the gene/chromosome ratio seems to be falling apart also in the case of *HER2*. A recent study of 1888 breast cancer patients treated with or without trastuzumab demonstrated that trastuzumab benefit was independent of *HER2*/centromere 17 ratio and Chr-17 copy number (Perez *et al*, 2010).
